# Modeling tool using neural networks for l(+)-lactic acid production by pellet-form *Rhizopus oryzae* NRRL 395 on biodiesel crude glycerol

**DOI:** 10.1186/s13065-018-0491-5

**Published:** 2018-11-29

**Authors:** Eva-H. Dulf, Dan Cristian Vodnar, Francisc-V. Dulf

**Affiliations:** 1 Automation Department, Technical University of Cluj-Napoca, Cluj-Napoca, Romania; 2 Food Science and Technology Department, University of Agricultural Sciences and Veterinary Medicine Cluj-Napoca, Cluj-Napoca, Romania; 3 Department of Environmental and Plant Protection, University of Agricultural Sciences and Veterinary Medicine Cluj-Napoca, Cluj-Napoca, Romania

**Keywords:** Software application, Neural network, Biodiesel, Predictive model

## Abstract

Most chemical reactions produce unwanted by-products. In an effort to reduce environmental problems these by-products could be used to produce valuable organic chemicals. In biodiesel industry a huge amount of glycerol is generated, approximately 10% of the final product. The research group from University of Agricultural Sciences and Veterinary Medicine Cluj-Napoca developed opportunities to produce l(+) lactic acid from the glycerol. The team is using the *Rhizopus oryzae* NRRL 395 bacteria for the fermentation of the glycerol. The purpose of the research is to improve the production of l(+) lactic acid in order to optimize the process. A predictive model obtained by neural networks is useful in this case. The main objective of the present work is to present the developed user-friendly application useful in modeling this fermentation process, in order to be used by people who are inexperienced with neural networks or specific software. Besides the interface for training of a new neural network in order to develop the model in some characteristic condition, the software also provides an interface for visualization of the results, useful in interpretation and as a tool for prediction.

## Introduction

Studies show that the increased usage of finite natural resources compels the search for a substitute. The most affected resource is considered to be the fuels: gas, petrol, etc. Bio-fuels have been developed for this purpose. Solving the search related problems new obstacles are created [1]. In the bio-chemical reaction which has as its product the bio-fuel, an unwanted by-product is created, glycerol. This organic substance is seldom used in other industries. Furthermore, it makes the quality of bio-diesel worse, caused by the big percentage of obtained glycerol (around 10% of the final product). The companies which produce the bio-diesel are bound to separate the products and need to handle the unwanted glycerol. This may result in the waste being thrown away, or in the better cases used to create a different organic substance. The synthesis of poly(glycerol-co-diacid) polyester materials is an attractive option for glycerol usage that can produce a wide range of products of commercial interest [2]. Biological based conversions are other attractive options, being efficient in providing products that are drop-in replacements for petro-chemicals and offer functionality advantage [3]. Another reconversion method of glycerol is the production of lactic acid, which has multiple uses in food, cosmetic and even pharmaceutics [4]. For industrial production of l(+)-lactic acid optimal conditions of fermentation, with higher yields and production rates must be developed, which can be obtained by bacterial fermentation [5]. After some experiments and research, the team from the University of Agricultural Sciences and Veterinary Medicine Cluj-Napoca concluded that the *R. oryzae* bacteria are the microorganism to use in their experiment with great results [6]. In order to optimize the fermentation process and to avoid time consuming, expensive experiments, the research team decided to develop an accurate mathematical model. The purpose of the model is to optimize the amount of resources used to create the l(+)-lactic acid. Since time and money are limiting factors, using them efficiently is necessary. A model can predict how the process can behave in shorter time and does not require any of the resources used for the reaction. However, it requires some experimental data which can be obtained by a limited number of experiments. In the presented paper the neural networks predictive method is used [7]. This modeling tool is inspired from the human brain cells. Neural networks excel at nonlinear processes due to their inherent properties. They have the ability to adapt and to learn, meaning sudden changes are less likely to affect them. The ability to generalize is one of the stronger points of this method, because it removes the limiting factor of the process.

In recent years, predictive models based on machine learning techniques have proven to be feasible and effective in modeling biochemical processes. However, to develop such a model, researchers usually have to combine multiple tools and must have strong programming skills to accomplish these jobs, which poses several challenges for users without advanced training in computer programming [8–11]. Therefore, an application that integrates all necessary steps for mathematical modeling of particular phenomena is a valuable and efficient solution that can meet the needs of related researchers and it is in continuous development.

The main objective of the present work is to develop a user-friendly application to model and predict the fermentation process from the production of l(+)-lactic acid, in order to be used by people who are inexperienced with neural networks or specific software. Besides the interface for training of a new neural network in order to develop the model, the software also provides an interface for visualization of the results, useful in interpretation and as a tool for prediction.

The structure of the work is the following. After the introductory part, “Results” section presents the developed application while “Discussion” section presents the results of a case study. Concluding remarks end the work.

## Results

The present application is constructed for the modeling and prediction stage of the fermentation process from the production of l(+)-lactic acid. In the experiments of the research team the variables are: the time, the concentration of glycerol and concentration of the Lucerne Green Juice used as supplement on media. The developed mathematical model has to establish the dependencies between the produced l(+) lactic acid and these variables. However, the same application, generalizing the labels, can be used in modeling any evolution which depends on three variables.

The developed application is based on use of neural networks. The main goal of the work was to make this application user friendly, not requiring knowledge in neural networks or some specific software.

The application is based on Matlab^®^ version R2016a [12]. To run the application, the user has to install the standalone application double-clicking “Applicenta”.

**Fig. 1:**
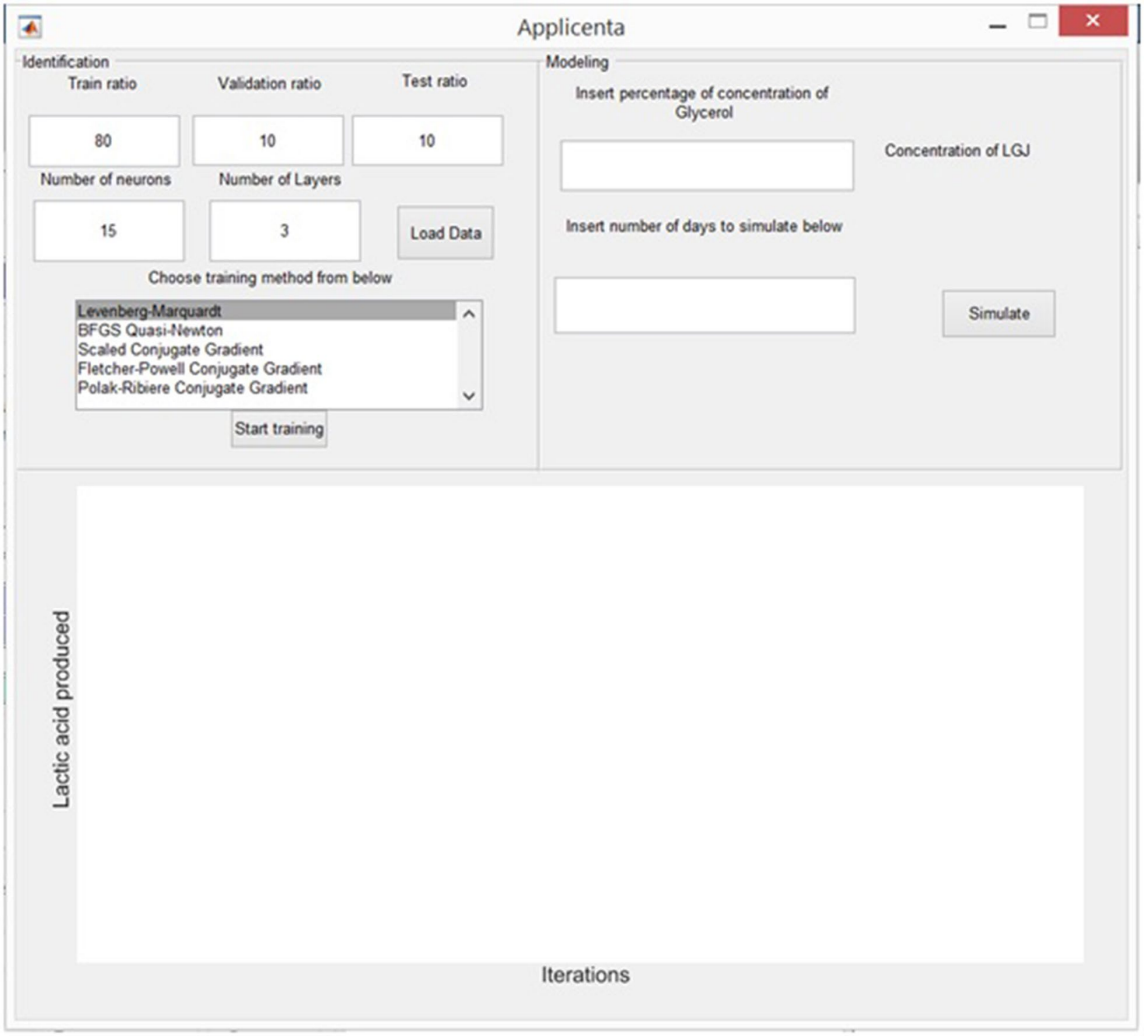
The application graphical user interface

The appearing graphical user interface is presented in Fig. 1. The application consist in three panels: the identification panel (upper left), the modeling panel (upper right) and the plotting panel (bottom panel) which is used by both identification and modeling panel.

### The identification panel

**Fig. 2:**
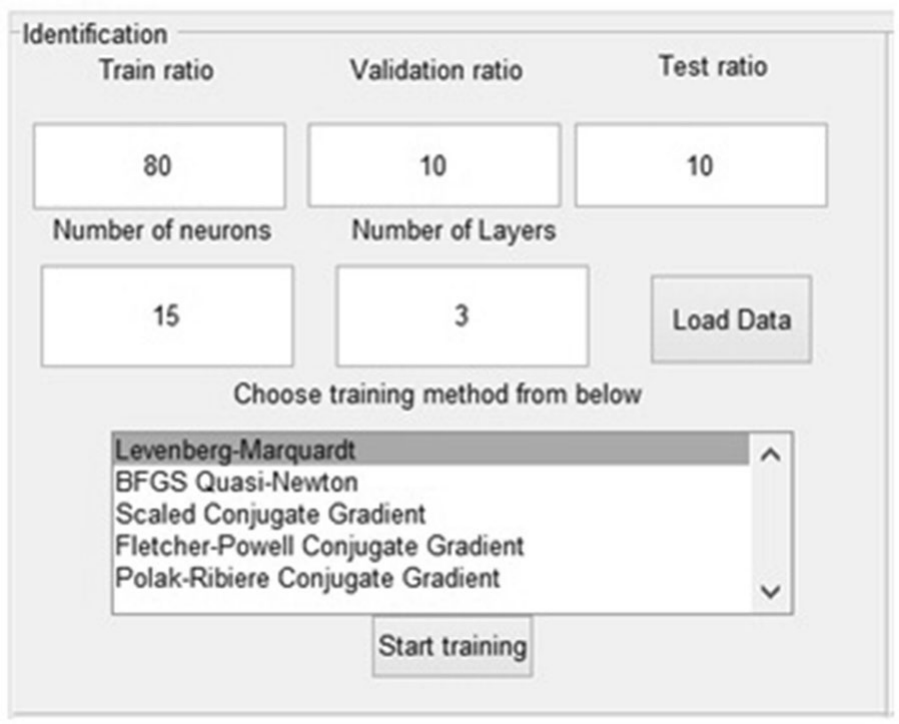
The identification panel

In this panel, presented in Fig. 2, the user can upload the experimental data and set the modeling conditions. The necessary steps to use it are described below.

Step 1: Import data. The experimental data you use for modeling must be saved in an excel file. This is loaded in the application with the press of the button called “Load Data”.
Step 2: Initialize the values which are going to be used in the training of the neural network. The number of layers and neurons are taken from the text boxes from the panel named “Number of Layers” and “Number of neurons” and their values are saved in two variables. The variables are used to create the hidden layer size for the neural network. These are one of the most important parameters, because they have the highest influence on the behavior of the model. Generally several trials are required to find the optimal values of these parameters. Increasing the number of layers and neurons lead to a large time computation.
Step 3: Choose the preferred ratios for training, validation and test, including the values in the text boxes called “Train ratio”, “Validation ratio” and “Test ratio”. Commonly the training ratio has the highest percentage, because the model is created with the amount of values given by this parameter. In a neural network it is important to have a high enough number of values in order to create the model. Having fewer values for training than for validation and testing leads to models with small accuracy. The other parameters, validation and testing, are for confirming whether the model is good or bad. The default percentages for the ratios are: 70% for training, 15% for validation and 15% for testing. In some cases, a higher number of values are required and the training ratio may be increased. Obviously, the sum of these three ratios must be 100 in order to use all the data you have.
Step 4: Choose the preferred algorithm. Each different training method has a different mathematic formula in its background. The name of the methods is also the name of the mathematic algorithm behind it. The training methods used in the application and experiments are: Levenberg–Marquardt (L–M), BFGS Quasi-Newton (Q-N), Scaled Conjugate Gradient (SCG), Polak–Ribiere Conjugate Gradient (P–R) and Fletcher Powell Conjugate Gradient (F–P). The user can freely choose which training method to use from the list box
Step 5: Start training by pushing the button called “Start training”.

**Fig. 3:**
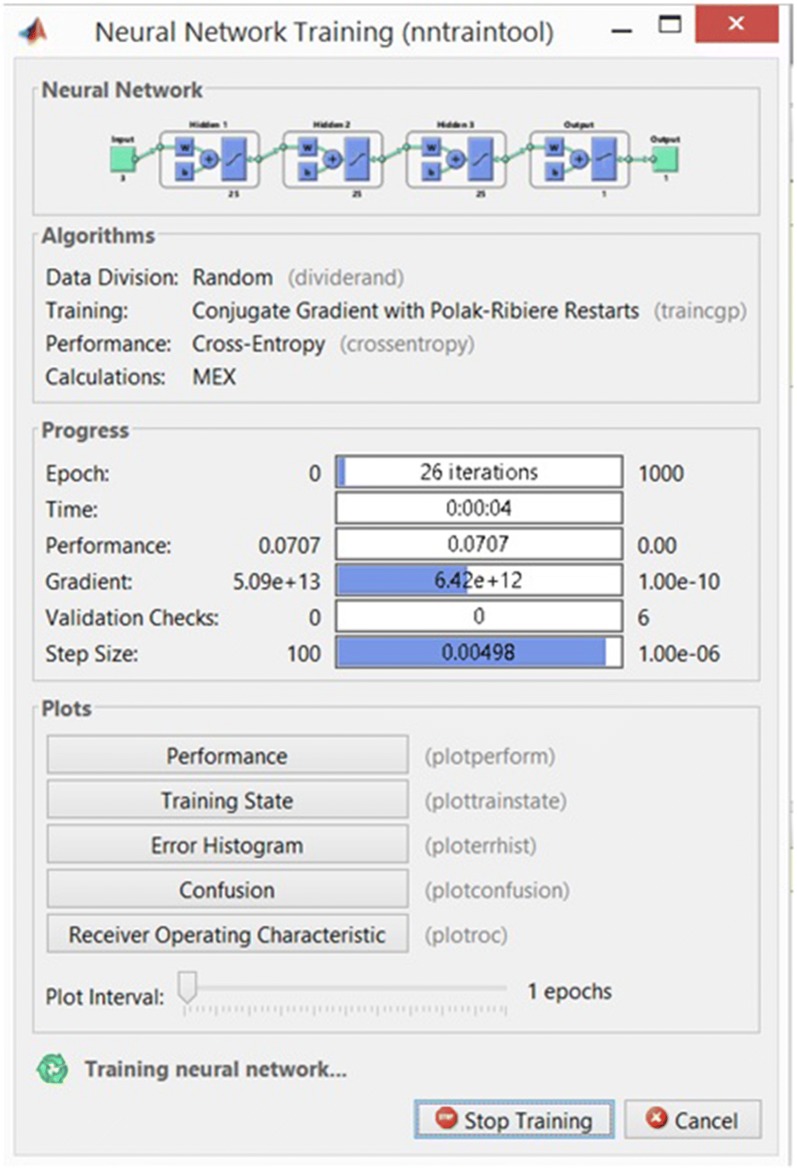
Neural network training

It appears a window like in Fig. 3, indicating the progress of the training stage.

**Fig. 4:**
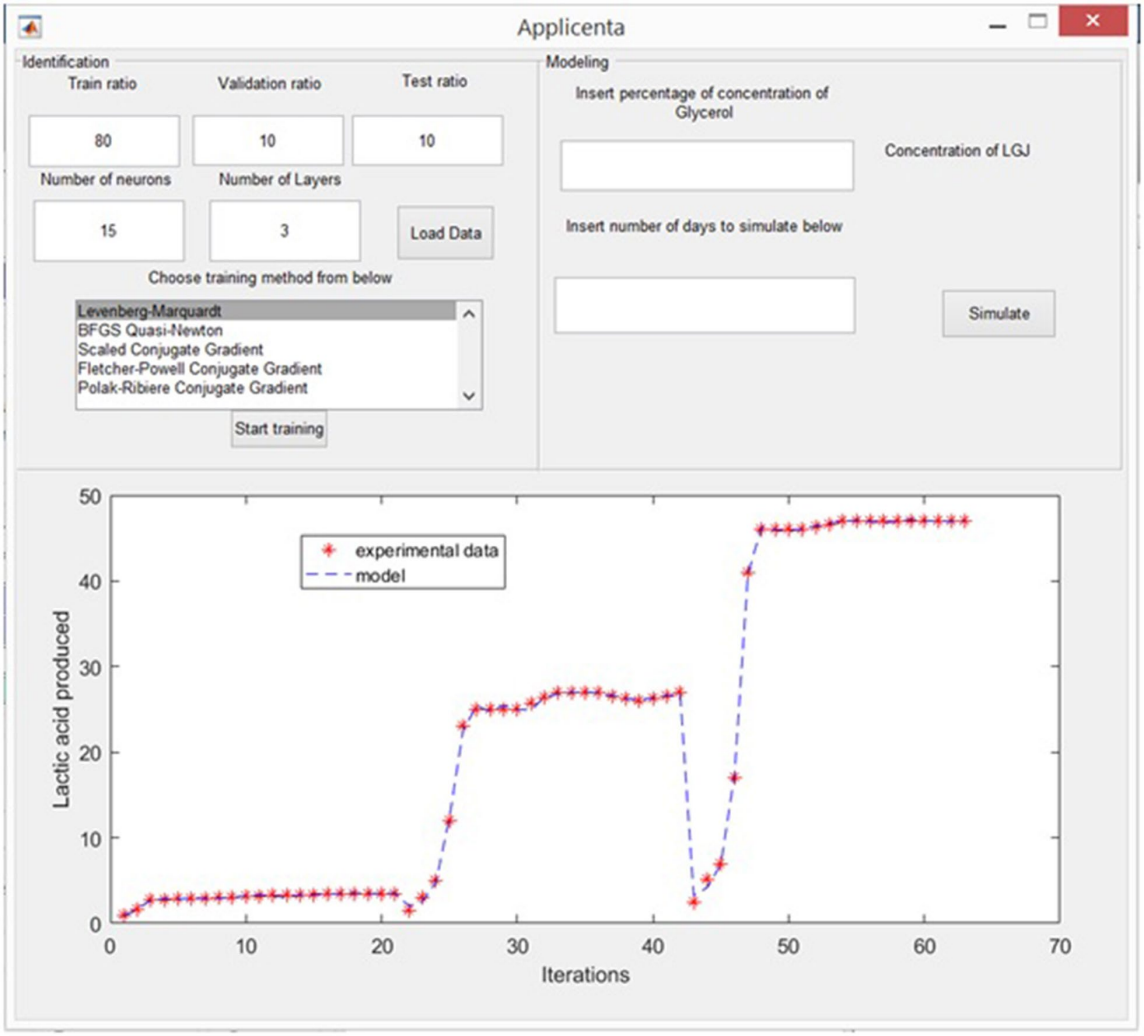
The results of the modeling stage

Finalizing the training stage, the predicted values in comparison with the experimental data are plotted in the bottom panel, Fig. 4. The user can decide if these results are satisfactory or not. If yes, it can proceed with the next stage, to predict some results for different conditions. If not, it may return to step 1 and choose different modeling conditions.

### The modeling panel

**Fig. 5:**
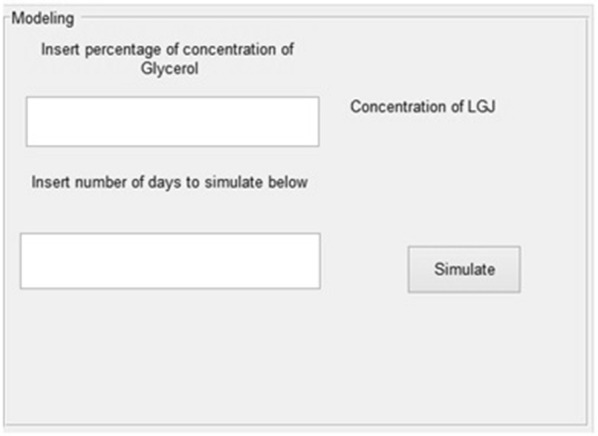
The prediction stage

With this panel, presented in Fig. 5, the user can obtain the predicted results for any values of the possible experimental conditions.

**Fig. 6:**
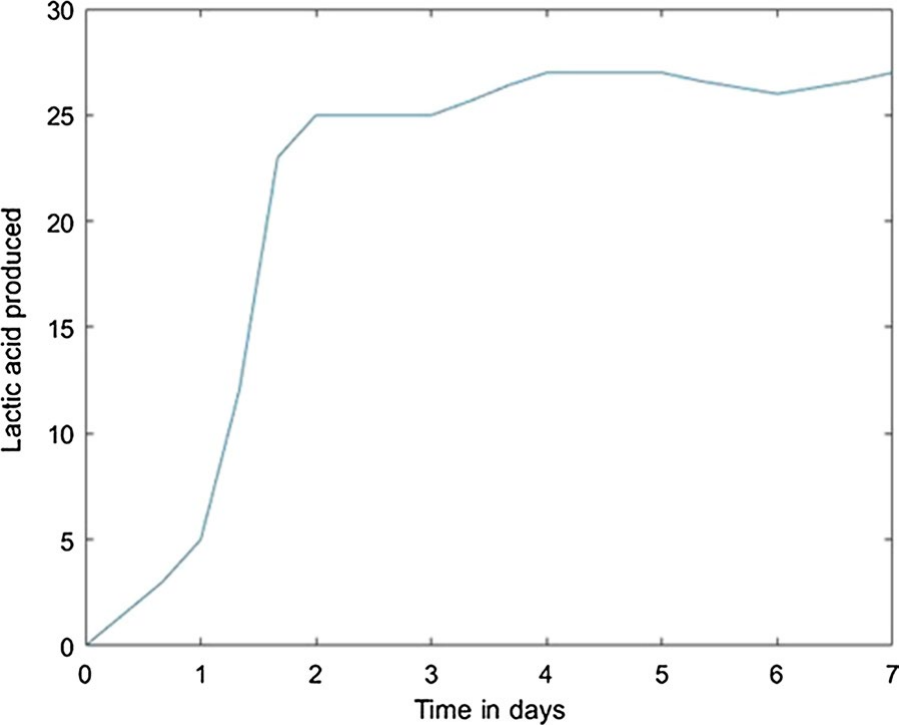
Predicted values based on the developed model

This panel requires the percentage of glycerol for which the simulation must be done and the number of days for which the virtual experiment should be executed. Using the model established on the previous stage, the predicted values will be plotted on the plot panel, Fig. 6. Of course, this prediction stage can be reloaded for any values the user whish.

## Discussion

In order to validate the developed tool, as case study were operated the experimental data from our previous publication [6].

**Fig. 7:**
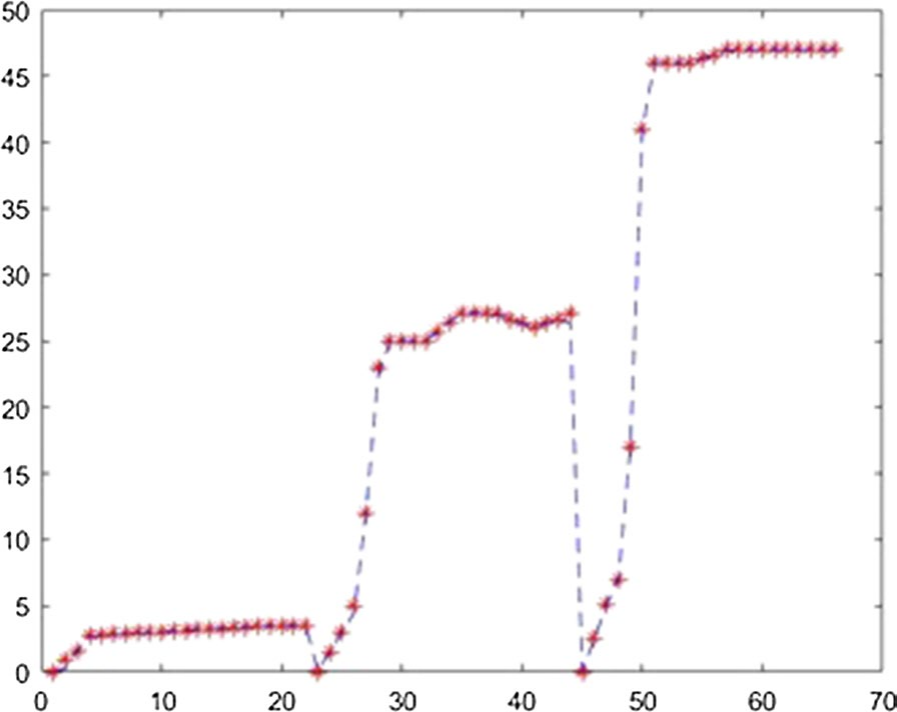
Model results obtained with Levenberg–Marquardt method

**Fig. 8:**
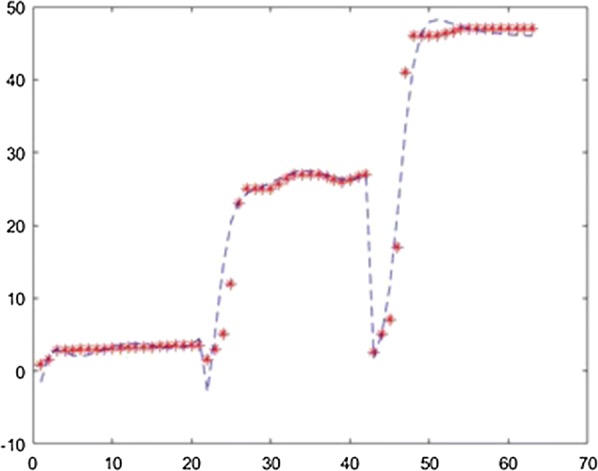
Model results obtained with the Quasi-Newton method

**Fig. 9:**
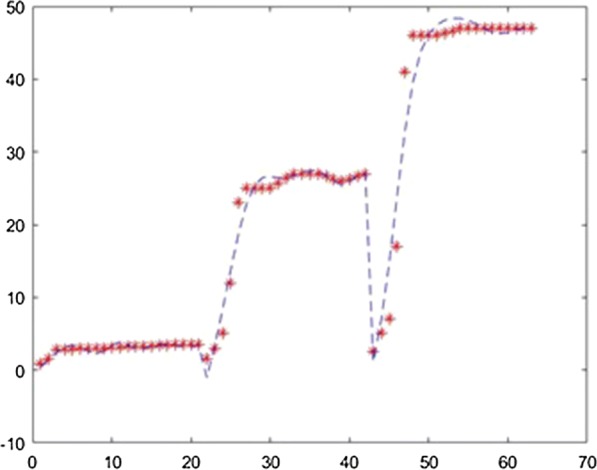
Model results obtained with the Scaled Conjugate Gradient method

**Fig. 10:**
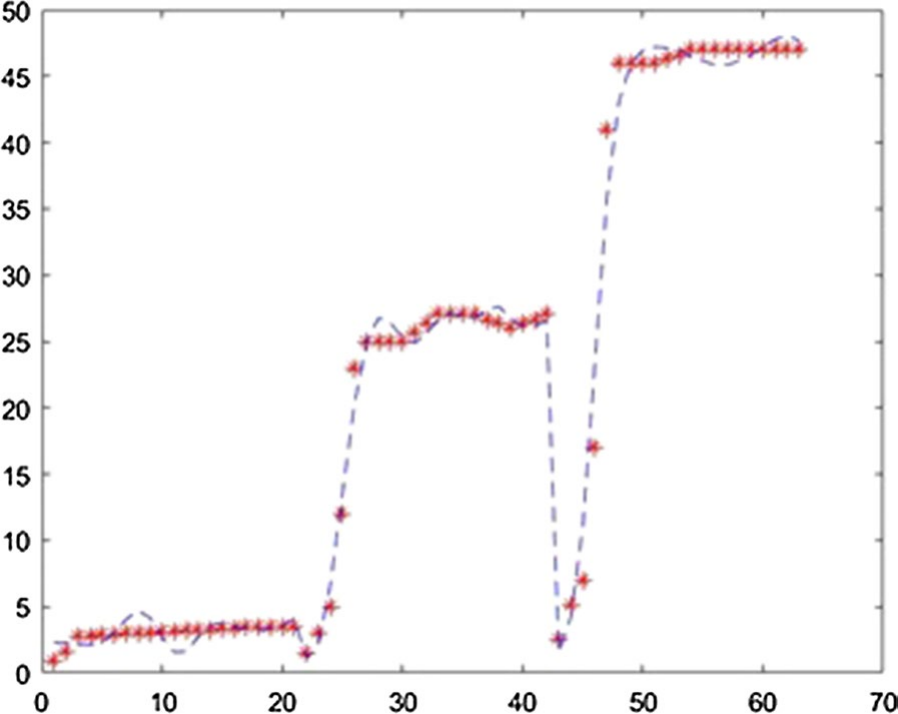
Model results obtained with the Fletcher–Powell Conjugate Gradient method

**Fig. 11:**
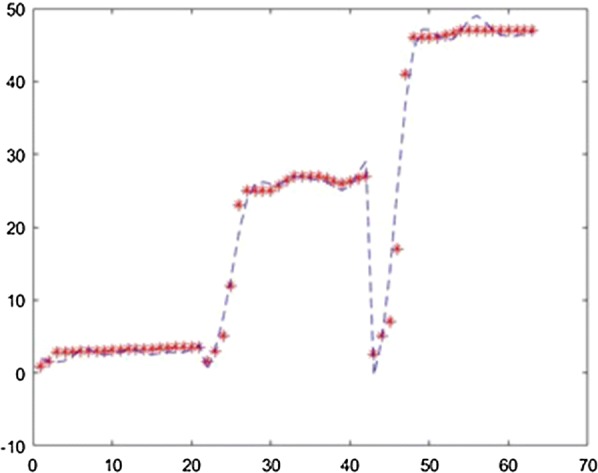
Model results obtained with the Polak Ribiere Conjugate Gradient method

The application was used to establish the model of the fermentation process from [6] with different neural network training methods. For each method the training ratio was chosen 80%, the validation ratio 10% and the test ratio 10%. The results obtained with 3 layers, with 25 neurons on each layer and using the Levernberg–Marquardt, Quasi-Newton, Scaled Conjugate Gradient, Fletcher–Powell Conjugate Gradient and Polak Ribiere Conjugate Gradient method are presented in Figs. 7, 8, 9, 10, 11.

**Fig. 12:**
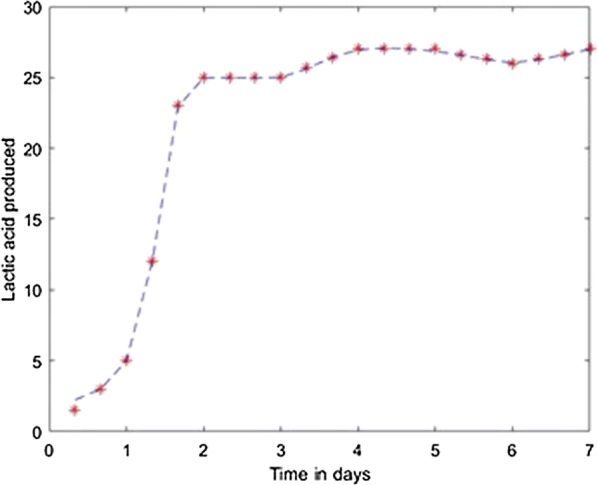
Simulation of the model on 40% glycerol 60% LGJ for Levenberg–Marquardt method

**Fig. 13:**
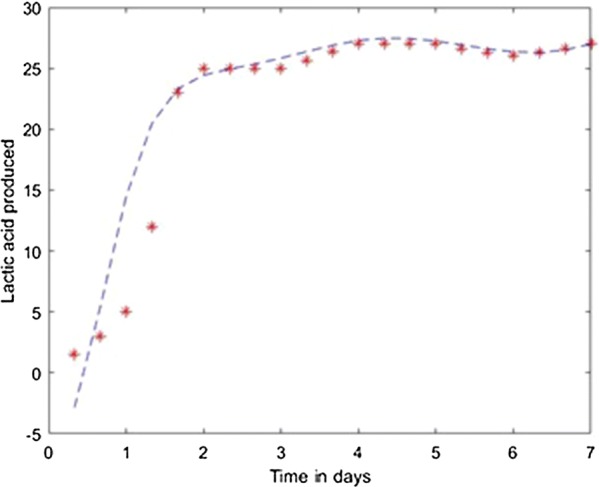
Simulation of the model on 40% glycerol 60% LGJ for Quasi-Newton method

**Fig. 14:**
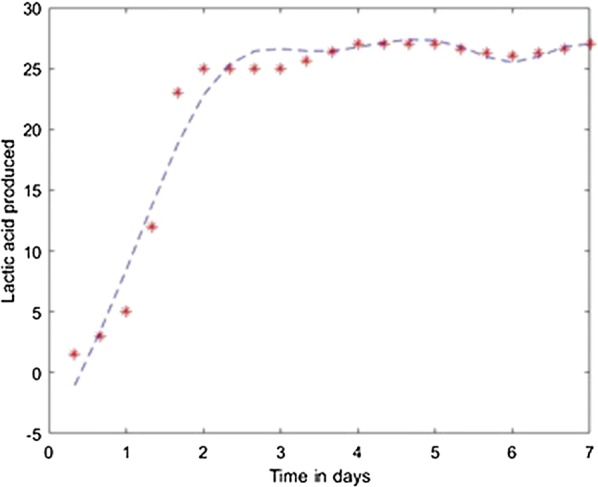
Simulation of the model on 40% glycerol 60% LGJ for Scaled Conjugate Gradient method

**Fig. 15:**
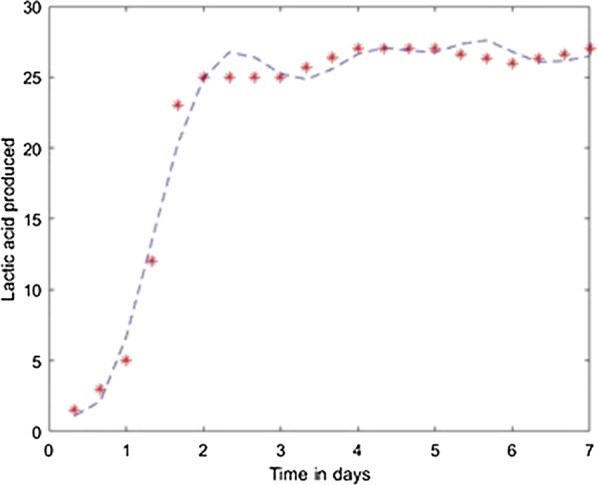
Simulation of the model on 40% glycerol 60% LGJ for Fletcher–Powell Conjugate Gradient method

**Fig. 16:**
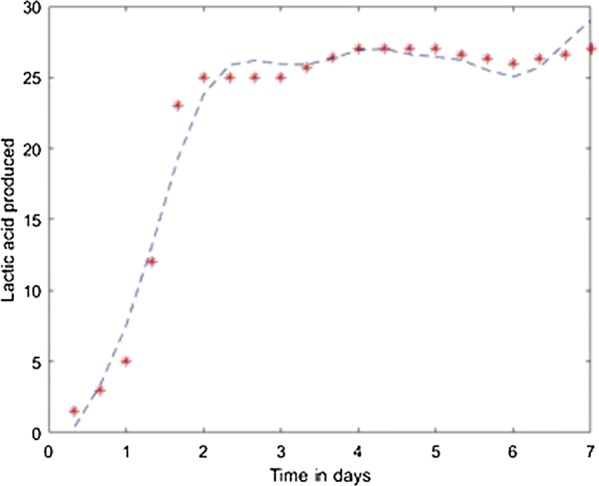
Simulation of the model on 40% glycerol 60% LGJ for Polak–Ribiere Conjugate Gradient method

For prediction stage, each resulted model was used to predict the l(+)-lactic acid production for 40% glycerol and 60% LGJ concentration for 7 days. The data corresponding to this case were not used in the modeling stage. The results, compared with experimental data, are presented in Figs. 12, 13, 14, 15, 16 for each method.

In order to compare the methods, the mean squared error was computed in each case, using different number of layers and neurons. These are presented in Table 1.

**Table 1:** Comparison of results

Training method	Number of layers	Number of neurons on each layer	Mean squared error
Levenberg–Marquardt	2	15	0.15
Levenberg–Marquardt	3	15	0.51
Levenberg–Marquardt	4	15	0.157
Levenberg–Marquardt	2	20	1.7
Levenberg–Marquardt	3	20	0.36
Levenberg–Marquardt	4	20	0.08
Levenberg–Marquardt	2	25	0.79
Levenberg–Marquardt	3	25	0.04
Levenberg–Marquardt	4	25	184.5
Quasi-Newton	2	15	244.23
Quasi-Newton	3	15	781.88
Quasi-Newton	4	15	482.86
Quasi-Newton	2	20	351.43
Quasi-Newton	3	20	499.8
Quasi-Newton	4	20	217.64
Quasi-Newton	2	25	431.66
Quasi-Newton	3	25	172.11
Quasi-Newton	4	25	898.75
Scaled Conjugate Gradient	2	15	244.23
Scaled Conjugate Gradient	3	15	781.88
Scaled Conjugate Gradient	4	15	482.86
Scaled Conjugate Gradient	2	20	351.43
Scaled Conjugate Gradient	3	20	499.8
Scaled Conjugate Gradient	4	20	217.64
Scaled Conjugate Gradient	2	25	431.66
Scaled Conjugate Gradient	3	25	172.11
Scaled Conjugate Gradient	4	25	898.75
Fletcher–Powell	2	15	244.23
Fletcher–Powell	3	15	781.88
Fletcher–Powell	4	15	482.86
Fletcher–Powell	2	20	351.43
Fletcher–Powell	3	20	499.8
Fletcher–Powell	4	20	217.64
Fletcher–Powell	2	25	431.66
Fletcher–Powell	3	25	172.11
Fletcher–Powell	4	25	898.75
Polak–Ribiere	2	15	244.23
Polak–Ribiere	3	15	781.88
Polak–Ribiere	4	15	482.86
Polak–Ribiere	2	20	351.43
Polak–Ribiere	3	20	499.8
Polak–Ribiere	4	20	217.64
Polak–Ribiere	2	25	431.66
Polak–Ribiere	3	25	172.11
Polak–Ribiere	4	25	898.75

In the present case study the Levenberg–Marquardt method proves the best fit with a least square error of 0.04 which is in accordance with the specific literature. The comparison of these algorithms—considering performance metrics like accuracy, sensitivity, specificity, etc.—concluded that the most efficient result can be achieved with Resilient Backpropagation and Levenberg–Marquardt algorithms [13]. It is also demonstrated that usually the fastest training algorithm is the Levenberg–Marquardt algorithm, but usually requires a lot of memory. That was the result in our case as well. The disadvantage of memory use is not relevant in our case, being an identification run on a performant computer and not on an edge hardware.

Another important conclusion of these results are that it demonstrates that increasing the number of layers and/or neurons do not lead to an automatic decrease of modeling error. This is also in accordance with the results provided in the literature. The number of layers and nodes are chosen based on experimentation, intuition and borrowed Ideas [14]. With equal training parameters (number of iterations, batch size, choice of optimizer), having a large number of layer can lead to high modeling error. The reason lies in back-propagation. The speed at which each layer learns is slower the further away it is from the output layer. Another reason for a possible high modeling error is that each layer is initialized randomly. If we don’t have enough data to train the effects of the randomness out, then we have the effect of the cumulative randomness.

## Conclusions

The most important strategy of biodiesel industry to overcome its productivity crisis and to reduce environmental problems is to produce valuable organic chemicals from by-products. For this purpose they have to focus on the by-product process optimization. Nowadays, machine learning based modeling approaches have been becoming a very popular choice to predict possible results without time and resource consuming experiments.

In this study, we developed an application to model and predict l(+)-lactic acid production by pellet-form *Rhizopus oryzae* NRRL 395 on biodiesel crude glycerol.

The main advantage of the proposed application is that it implements a complete online model-building process, which enables biochemical researchers to construct predictive models easily without suffering from tedious programming and deployment work.

## References

[1] Oh YK, Hwang KR, Kim C, Kim JR, Lee JS (2018). Recent developments and key barriers to advanced biofuels: a short review. Bioresour Technol.

[2] Valerio O (2018). Poly(glycerol-co-diacids) polyesters: from glycerol biorefinery to sustainable engineering applications, a review. ACS Sustain Chem Eng.

[3] Pradima J (2017). Review on enzymatic synthesis of value added products of glycerol, a by-product derived from biodiesel production. Resour Effic Technol.

[4] de Oliveira RA, Komesu A, Rossell CEV, Maciel Filhoa R (2018). Challenges and opportunities in lactic acid bioprocess design—from economic to production aspects. Biochem Eng J.

[5] Ilmén M, Koivuranta K, Ruohonen L, Rajgarhia V, Suominen P, Penttilä M (2013). Production of l-lactic acid by the yeast *Candida sonorensis* expressing heterologous bacterial and fungal lactate dehydrogenases. Microb Cell Fact.

[6] Vodnar D (2013). l(+)-lactic acid production by pellet-form *Rhizopus oryzae* NRRL 395 on biodiesel crude glycerol. Microb Cell Fact.

[7] Suryawanshi B, Mohanty B (2018). Application of an artificial neural network model for the supercritical fluid extraction of seed oil from *Argemone mexicana* (L.) seeds. Ind Crops Prod.

[8] Dulf FV, Vodnar DC, Dulf EH, Pintea A (2017). Phenolic compounds, flavonoids, lipids and antioxidant potential of apricot (*Prunus armeniaca* L.) pomace fermented by two filamentous fungal strains in solid state system. Chem Cent J.

[9] Raškevičius V, Mikalayeva V, Antanavičiūtė I, Ceslevičienė I, Skeberdis VA, Kairys V, Bordel S (2018). Genome scale metabolic models as tools for drug design and personalized medicine. PLoS ONE.

[10] Wang X, Jiang Y, Hu D (2016). Optimization and in vitro antiproliferation of *Curcuma wenyujin’s* active extracts by ultrasonication and response surface methodology. Chem Cent J.

[11] Abdollahi Y (2013). Artificial neural network modeling of *p*-cresol photodegradation. Chem Cent J.

[12] https://mathworks.com/. Accessed 25 June 2018

[13] Cömert Z, Kocamaz AF (2017). A study of artificial neural network training algorithms for classification of cardiotocography signals. J Sci Technol.

[14] Goodfellow I (2016). Deep learning (adaptive computation and machine learning).

